# Role of Vitamin D in Athletes and Their Performance: Current Concepts and New Trends

**DOI:** 10.3390/nu12020579

**Published:** 2020-02-23

**Authors:** Mirian de la Puente Yagüe, Luis Collado Yurrita, Maria J. Ciudad Cabañas, Marioa A. Cuadrado Cenzual

**Affiliations:** 1 Department of Public and Maternal Child Health University, School of Medicine, Complutense University of Madrid, 28040 Madrid, Spain; miriampu@ucm.es; 2 Department of Medicine, School of Medicine, Complutense University of Madrid, 28040 Madrid, Spain; mjciudad@ucm.es (M.J.C.C.); macuad02@ucm.es (M.A.C.C.)

**Keywords:** vitamin D, athletic performance, 25(OH)D, supplementation, deficiency, athlete

## Abstract

We are currently experiencing a vitamin D (VITD) deficiency pandemic across the world. Athletes have the same predisposition to low levels of vitamin D, the majority of its concentrations being below 20 ng/mL in a wide range of sports, especially in the winter months. Vitamin D is important in bone health, but recent research also points out its essential role in extraskeletal functions, including skeletal muscle growth, immune and cardiopulmonary functions and inflammatory modulation, which influence athletic performance. Vitamin D can also interact with extraskeletal tissues to modulate injury recovery and also influence the risk of infection. The data presented in this paper has triggered investigations in relation to the importance of maintaining adequate levels of vitamin D and to the possible positive influence supplementation has on immune and musculoskeletal functions in athletes, benefiting their performance and preventing future injuries. The objective of this review is to describe the latest research conducted on the epidemiology of vitamin D deficiency and its effects on sports performance and musculoskeletal health.

## 1. Introduction

Over the past decade, interest in research in relation to vitamin D (VITD) has been growing exponentially, partly due to the increased prevalence of its deficiency in the population and the association between the deficiency of VITD and a wide range of diseases [1,2,3]. The importance and versatility of vitamin D in the organism is becoming increasingly evident. VITD plays an active role in immune function, protein synthesis, muscle function, cardiovascular function, inflammatory response, cell growth and musculoskeletal regulation [2,3,4,5]. 

In relation to vitamin D and its role in athletes, an important field of research on its influence on bone balance, muscle resistance and athletic performance is currently underway [6,7,8,9,10].

A priori, athletes might seem to have sufficient levels of VITD. However, the latest research shows that this assumption is wrong. In the last decade, the scientific community has conducted studies on VITD levels in various groups of athletes including runners, basketball players, jockeys, gymnasts and even dancers, showing that these levels in athletes are comparable to those of the general population. However, recent publications show that these levels will considerably depend on geographical location, and on the type of sport, whether it is indoor or outdoor, etc. A line of special interest is the influence of VITD deficiency on athlete morbidity [8,9,10,11,12]. The deficiency of this vitamin is generally widespread in the athletic population with an increase in morbidities associated with it, and the appearance of osteomalacia and osteoporosis [10,11,12]. Given the high prevalence of its deficiency and its negative potential on morbidity, the possible determination of VITD levels in athletes is considered part of the screening routine [7,10].

In relation to VITD supplementation in athletes with deficiency, several studies have shown that this increases muscle strength. Higher serum levels of vitamin D are associated with reduced injury rates and better sports performance. It is important to correctly identify people with vitamin D deficiency who need supplements to help optimize their performance and prevent future injuries [1,10,11].

Finally, it seems that there is a paradoxical relationship between ethnicity and VITD concentration. As an example, white-skinned subjects generally have lower levels of VITD but higher bone mineral density and decreased risk of fracture [6,12].

This review was prepared by searching available medical and scientific literature from PubMed, EMBASE and Cochrane Library. Nutrition, endocrinology, biochemistry, orthopedics, sports and toxicology journals, among others, were analyzed as well as by reviewing several books, conference proceedings, government publications.

## 2. Synthesis and Metabolism of Vitamin D 

On the one hand, VITD is a micronutrient, since its deficiency can be treated by supplementation, and it is also a prohormone, seeing that its precursors are transformed into active metabolites. It comes in two biologically inactive forms, cholecalciferol (vitamin D3) and ergocalciferol (vitamin D2) [2,4,13].

Vitamin D is mostly synthesized in the skin. Cholecalciferol, or vitamin D3, is the primary source of endogenous VITD and is formed through the interaction of ultraviolet B (UVB) radiation after sun exposure with 7-dehydrocholesterol, which is stored inside the plasma membrane of every skin cell. Ergocalciferol, or vitamin D2, represents a small percentage and has its origin in exogenous dietary intake [14,15,16]. Vitamin D is difficult to obtain through diet because very few foodstuffs contain the vitamin naturally, the exceptions being the liver of fatty fish, mushrooms and eggs, among others. Supplementation or fortification with vitamin D2 and D3, such as milk and other dairy products, cereals, etc., currently implies an exogenous supply [14,16].

The VITD obtained from sun exposure, food or supplementation is biologically inert and must undergo two hydroxylations in the organism to become active, the first being performed in the liver by the CYP2R1 enzyme where it is converted to 25-hydroxyvitamin D3 (calcidiol). The second being performed in the kidney and other tissues by the CYP27B1 enzyme to form 1.25-dihydroxyvitamin D3 (calcitriol) that is the biologically active form. The active metabolite of vitamin D is transported through the bloodstream by the binding protein vitamin D (BPD), reaching numerous skeletal and extraskeletal target organs. In fact, the CYP27B1 enzyme is present in many target cells in the body to allow local synthesis of calcitriol. In addition, vitamin D receptors (VDR) are present in most tissues [15,16,17,18]. 

**Figure 1:**
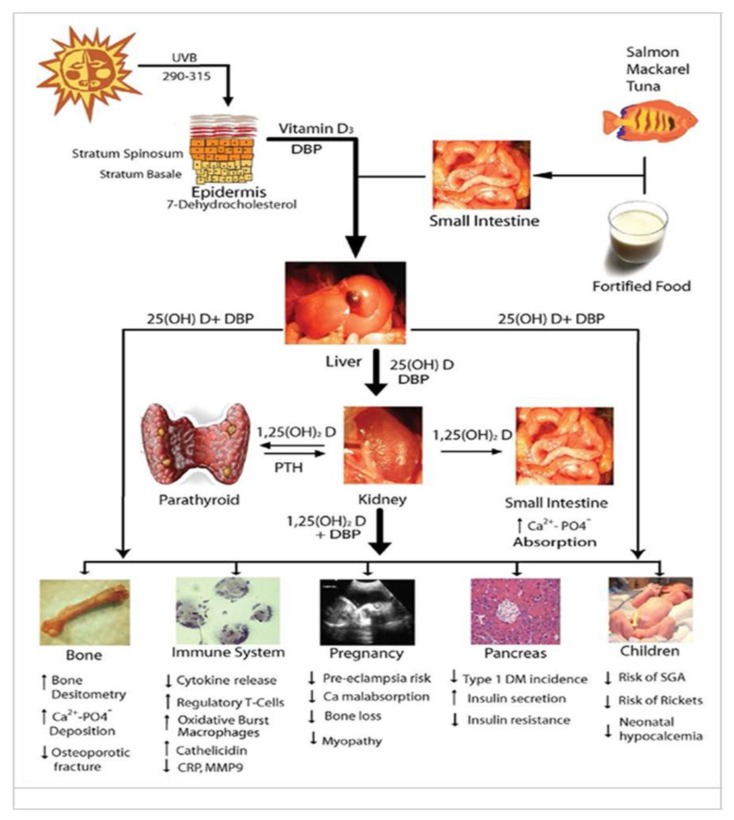
Vitamin D metabolism and its action in the body. Source of Figure 1: Mulligan, M.L.; Felton, S.K.; Riek, A.E.; Bernal - Mizrachi, C. Implications of vitamin D deficiency in pregnancy and lactation. Am J Obstet Gynecol 2010, 202 (5), 429 [14]. PTH (Parathyroid Hormone). DPB (Vitamin D-Binding Protein) DM (Diabetes Mellitus) SGA (Small for Gestational Age).

The metabolism of VITD and its functions in different systems of the human body are shown in Figure 1 [14]. 

## 3. Vitamin D Mechanism of Action

The functions of VITD are performed in the body via two pathways through endocrine and autocrine mechanisms [19,20,21,22,23]. The endocrine mechanism is the most studied and works by increasing intestinal calcium absorption and osteoclastic activity. Vitamin D is essential in bone growth, density and remodeling [13,18,21,22,23]. When vitamin D levels decrease below normal limits, PTH increases bone resorption to meet the body’s demands for calcium. This means that low levels of VITD lead to an increase in bone turnover with an added risk of bone injury such as stress fractures, which are very common in athletes.

The second mechanism of action of vitamin D involves an autocrine pathway. Although it is not so well known, this pathway is essential since it hosts many of the organism’s key metabolic processes, such as signaling processes, expression and genetic response, hormone protein synthesis, immune/inflammatory response, turnover and cell synthesis. Without VITD, the ability to effectively respond to physiological and pathological symptoms would be totally altered [19,20,21,22]. This vitamin works as a modulator of up to 2000 genes involved in cell growth, immune function and protein synthesis [15,21,23].

The autocrine pathway seems to be the most important in relation to the action of vitamin D on skeletal muscle function. Targets for the VITD receptor have been identified in almost every body tissue. VDR regulates expression in hundreds of genes that perform essential bodily functions. The discovery of VDR in muscle suggests the importance of the role of VITD in muscle tissue [19,20,21,22,23]. 

At present, the existing theory is that an adequate concentration of vitamin D in the blood is necessary to optimize the function of genomics [9]. This role as a genetic modulator explains how vitamin D can affect a variety of physiological functions, such as bone health, muscle function, inflammation and immunity, all important for health, training and performance [19,21].

In the article published by Owens and collaborators in 2016 [4], there is an excellent schematic representation in which the above can be observed [4].

## 4. Prevalence of Deficiency and Insufficiency of Vitamin D in Athletes

Not only is it estimated that 1 billion people in the world currently have VITD deficiency, but the progressive increase in its prevalence worldwide is also worrying [5,9,24,25,26,27,28]. Most articles evidence that VITD deficiency is widespread across the world and at prevalence rates that meet the criteria of a pandemic (definition of a pandemic: “an epidemic occurring worldwide, or over a very wide area, crossing international boundaries and usually affecting a large number of people”) [5,14,25]. However other authors questioned this sentence [28]. VITD deficiency is a frequent finding among the American population, so much so that 36% to 57% of adults are deficient [27,28,29]. This deficiency is also common in Europe mostly countries in Northern European latitudes (>35° N) such as the UK, Ireland, Denmark, France, Germany, etc. [5,25,29]. A similar prevalence has been found even in areas where there is greater sun exposure, such as Australia, the USA and Saudi Arabia [8,27,28,29]. In Canada, 30–50% of children and adults have VITD deficiency. Similar data has been found in other countries, see Africa, New Zealand, Brazil, etc., evidencing a high risk of VITD deficiency in both adults and children [24,25,27,29]. 

The main factors for VITD deficiency are cultural and environmental influences. The major cause for the VITD deficiency pandemic is the lack of awareness of the population that sun exposure is the main source of vitamin D. In relation to food sources of VITD, it is difficult to obtain vitamin D through the diet because very few foods naturally contain the vitamin, exceptions being the liver of fatty fish such as salmon, sardines, herring and red meat. Actually, diet source includes fortified foods such as milk, fat spreads and cereals. Due to the critical role played by exposure to sunlight and, in particular, to ultraviolet radiation in the synthesis of VITD, any factor that alters this mechanism will contribute to VITD deficiency, such as the decrease of UVB radiation reaching the earth’s surface, the use of sunscreens, melanin that diminishes the effectiveness of sun in producing VITD, polluting atmospheric particles, latitude, weather, lifestyles, etc. [8,9,12,19,22,24,25,29]. In addition, numerous endogenous factors can alter the production of vitamin D and can induce its deficiency, such as its altered metabolism, malabsorption or insufficient intake in one’s diet [24,25,29].

Current strategies in Public Health include dietary supplementation with VITD and education of young children and adolescents. Such initiatives have an important effect on the decrease in the prevalence of developmental problems such as rickets and stunting [2,3,5,8,9]. Other strategies support no need to perform screening everyone for the VITD status. It is more cost-effective to increase food fortification with VITD [29]. However, symptoms of VITD deficiency in adults, osteoporosis, osteomalacia and immune deficiencies are ignored in most cases. Patients with VITD deficiency have musculoskeletal pains that are often misdiagnosed as fibromyalgia, chronic fatigue syndrome and myositis, among others [30,31,32,33,34,35,36,37]. 

In relation to athletes, VITD deficiency within the global athletic population also follows the same patterns [7,10,12,30,31,32,33,34,35,36,37]. When VITD levels in professional athletes are analyzed, we observe that they are all affected in a similar way. The different studies present the following results: among basketball professionals, 32% of the athletes were found to be deficient and 47% had VITD insufficiency. Among National American Football League players, 26% had VITD deficiency and 42% to 80% showed levels of insufficiency. Of Liverpool’s professional football players, 36% showed deficiency or insufficiency [30,31,32,33,34,35,36]. 

Deficiencies or insufficiencies have been found in most dancers, swimmers, volleyball players, taekwondo fighters, jockeys, runners, weightlifters, etc. [31,32,33,34,35,36,37]. 

Furthermore, multiple studies have shown that dark-skinned athletes have a higher risk of suffering from secondary alterations due to VITD deficiency [26,28,34,35]. One study showed that athletes with high concentrations of melanin in their skin need up to 10 times longer exposure to ultraviolet (UVB) radiation to generate the same reserves of VITD as light-skinned athletes. A study by Mehran et al. on professional hockey players in which vitamin D deficiency was 0% and insufficiency only appeared as 13% should be noted. The authors attributed this low frequency to race, since 96.2% of the players were Caucasians [34,37]. 

In relation to the degree of solar exposure and athleticism, the distance to the equator, season and weather will dictate the source of solar VITD. The production of VITD from the solar source will obviously be influenced by hours of sunshine, pollution, sun block, skin pigment, age, etc. During the summer months and/or countries with more hours of sunshine, UVB radiation from the sun can be absorbed in sufficient amounts to synthesize VITD [4,6,19,32]. However, during the winter months, the angle of the sun prevents UVB radiation from reaching latitudes above 35–37 degrees. When analyzing levels of VITD in athletes, it should be taken into account that these can vary according to the season, place of training, type of sport and skin color [4,6,30,35].

According to some authors, the levels of vitamin D are generally lower in the winter months [30,31,33,34]. However, suboptimal vitamin D levels occur even in sunny countries near the equator when the sun is avoided or the skin is protected. Despite all the factors mentioned above, a high prevalence of vitamin D deficiency has been documented in athletes in both outdoor and indoor sports [3,6,25,35,36,37]. A recent meta-analysis that groups together 23 studies composed of 2313 athletes found that 56% had insufficient levels of vitamin D [23]. Koundourakis et al. [11,12] showed that professional Greek football players who trained at a latitude of 35.9° did not have insufficient levels of vitamin D. Almost identical levels were reported in players of the National Football League, in elite gymnasts in Australia and in young Hawaiian skaters and a variety of other athletes around the world. These findings were observed regardless of sun exposure. In a recent study conducted in Israel at a favorable latitude (31.8° N) for sunshine, 73% of athletes were deficient in vitamin D [1,3,6,25,35,37].

Finally, in relation to dietary recommendations, studies find that athletes do not come close to meeting these in most countries. One study found that only 5% of college athletes met the US Recommended Dietary Allowance (RDA) [32].

## 5. Assessment of Vitamin D Status Determination of 25-OHVITD

Status of levels of insufficiency or deficiency of VITD can be defined by using an indicator to determine the blood level of total 25-hydroxy vitamin D (25-OHVITD). This indicator is currently considered as the most qualified to show the body store of vitamin D [38,39,40].

The measurement of blood levels of 25-OHVITD will show us the cutaneous production of VITD that is obtained from food and supplements. It should be noted that the plasma average life is approximately 15–20 days and is recognized as a biomarker of exposure. However, it is controversial whether blood levels of 25-OHVITD could be considered as a biomarker of effect (e.g., relationship with the state of health, etc.) since serum 25-OHVITD levels do not indicate the amount of VITD stored in body tissues [40,41,42].

Unlike 25-OHVITD, the determination of 1.25 dihydroxy vitamin D (1.25 (OH)2 VITD) is generally not a good indicator of VITD levels, since it has a very short average life (barely 15 h) and its serum concentrations are closely regulated by PTH calcium and phosphate. In fact, levels of 1.25 (OH)2 VITD do not drop significantly until a severe deficiency of VITD occurs [38,39].

The latest research is aimed at being able to measure the unbound fraction of 25-OHVITD, or rather, the fraction of VITD that is not bound to plasma proteins and that exerts biological activity. The unbound form can pass through the cell membrane and, therefore, carry out its function [42,43,44].

After several years of research, a new method was developed in 2017 that allows the concentration of unbound 25-OHVITD to be measured. This method measures the concentration of the unbound fraction, based on an enzyme-linked immunosorbent immunoassay (ELISA). The separation of unbound and bound forms, as well as the capture of the former, is achieved through the use of a monoclonal antibody (anti-25-OHD), disrupting as little as possible the balance between both forms [44]. Following the appearance of this method, new investigations are emerging, although the usefulness of measuring unbound 25-OHVITD has yet to be established in normal clinical practice.

## 6. Levels and Classification of Vitamin D Levels

In the past decade, there has been an exponential increase in the prevalence of deficiency in the population, and in some studies, it is claimed that we are facing an emerging epidemic situation in relation to low blood levels of 25-OHVITD. From the above, it can be clearly deduced that it is extremely important to adequately define the status of deficiency and insufficiency and optimal levels of vitamin D in the population. The definition of VITD levels for its classification has traditionally been very controversial. At present, it is suggested that its establishment should be based on levels and on clinical and disease risk markers [5,9,45,46,47,48]. Some authors propose that the clinical ranges of vitamin D need to be based on the association of 25-OHVITD deficiency, osteomalacia, rickets and the approximate concentration at which PTH rises sharply. On the other hand, it is proposed that the limit for insufficiency should be the concentration at which the PTH plateau and calcium absorption are maximized [5,9,45,48].

There are studies which suggest that a value of 25-OHVITD >30 ng/mL should be considered as acceptable for maintaining bone health and reducing the risk of fracture in healthy young people and adults, while others suggest that necessary levels should be set at >40 ng/mL [3,8,25,45]. On a more conservative basis, the US Institute of Medicine (IOM) states that concentrations of ≥20 ng/mL (50 nmol/L) should meet the needs of 97.5% of the population [5,9,14,29]. The IOM also establishes an inappropriate level of VITD when levels are between 12 and 20 ng/mL (30 and 50 mmol/L), and finally, people are at risk of VITD deficiency when their levels are below 12 ng/mL (30 nmol/L). Serum concentrations above 125 nmol/L (>50 ng/mL) are associated with potential adverse effects, and finally, levels above 150 ng/mL should be considered as toxic [47]. Unfortunately, there are currently no precise thresholds to classify the condition of athletes although Close et al. argue that those athletes with serum levels of 25-OHVITD below 12 ng/mL should be considered for supplementation, in accordance with IOM guidelines [32] (Table 1).

## 7. Role of Vitamin D and Its Relationship with the Condition of the Athletes

### 7.1. Effect of Vitamin D on Calcium Homeostasis and Bone Balance

Traditionally, it has been assumed that the main function of vitamin D was the maintenance of calcium homeostasis and serum phosphate. An adequate amount of vitamin D and calcium is required for the development, growth and integrity of bones. Currently, VITD has been shown to influence bone health by activating the expression of genes that improve intestinal absorption and renal reabsorption of calcium (in association with an increase in PTH) and bone turnover [18]. Vitamin D also contributes to the mobilization of calcium of the bone by means of osteoclastogenesis that results from the activation of several genes, including the activator of the K-ligand nuclear factor receptor (RANKL) and the RANKL system [3,5,49,50,51].

On the other hand, VITD is closely related to the parathyroid hormone (PTH). Together, these hormones closely regulate the concentration of calcium in the serum. Chronic VITD deficiency leads to secondary hyperparathyroidism. This combination of vitamin D deficiency and an elevated PTH level can cause excessive mobilization of calcium from the bone to maintain circulating calcium levels at the expense of bone mineral density [51,52,53,54,55].

Furthermore, research suggests that the concentration of VITD in the blood is associated with bone mineral density (BMD) and/or mineral content in the hip and lumbar vertebrae of women throughout their lives [51,53]. Current literature shows inconsistent associations between bone mineral density (BMD) and vitamin D levels [51,52], particularly in racial minorities and athletic populations. The load stimulus to which the musculoskeletal system is subjected through a high-intensity dynamic sports activity is believed to compensate for 25-OHVITD deficiency and prevent poor bone health in athletes. However, Hamilton et al. demonstrated that BMD and the level of 25 [OH] D were not statistically linked in a study conducted in male athletes from the Middle East, suggesting that genetic polymorphism in path 25 [OH] D/1.25 [OH] D can explain some of these differences. While it is considered that athletes should have “sufficient” vitamin D concentrations to optimize bone mineral density (BMD), the exact value to “optimize” bone health is still unclear [53].

Finally, vitamin D also increases the activity of the insulin-like growth factor 1 (IGF-1) through induction of its receptor expression, which has a crucial effect on bone formation both in vitro and in vivo [55,56].

### 7.2. Effect of Vitamin D on Fractures

A particularly relevant section refers to the action of VITD in stress fractures, which are frequently observed in athletes, which represent from 0.7% to 20% of all clinical injuries in sports medicine. Although it has already been stated that vitamin D levels can affect BMD, there is less knowledge of the role of vitamin D in fracture healing and there is no scientific evidence on it. A review found that vitamin D reduces, increases or has no effect in the soft callus formation phase during the fracture healing process [54]. Other studies find conflicting results regarding the effect of vitamin D in the callus mineralization phase [55]. However, a recent investigation found lower serum levels of 25-OHVITD in patients with delayed fracture consolidation, while other studies found no differences between patients with diaphyseal fractures and those who presented delayed healing [56].

### 7.3. The role of Vitamin D in the Skeletal Muscle

Vitamin D has been shown to be a powerful modulator of skeletal muscle physiology [57,58,59,60]. Vitamin D influences it by activating the expression of genes that influence muscle growth and differentiation, particularly in fast-twitch fibers (type II) [60,61,62]. In addition, enlarged interfibrillar spaces and infiltration of fat, fibrosis and glycogen in muscular dystrophies are shown in muscle biopsies of individuals with VITD deficiency [62]. Biopsies of 12 patients with VITD deficiency, before and after treatment with the vitamin, found atrophy of type 2 muscle fibers before treatment and significant improvement after it [61].

It should be noted here that both genomic and non-genomic effects of VITD are crucial for muscle performance. In fact, vitamin D affects calcium and phosphate transport by muscles through cell membranes, phospholipid metabolism and muscle cell proliferation and differentiation [62].

**Figure 2:**
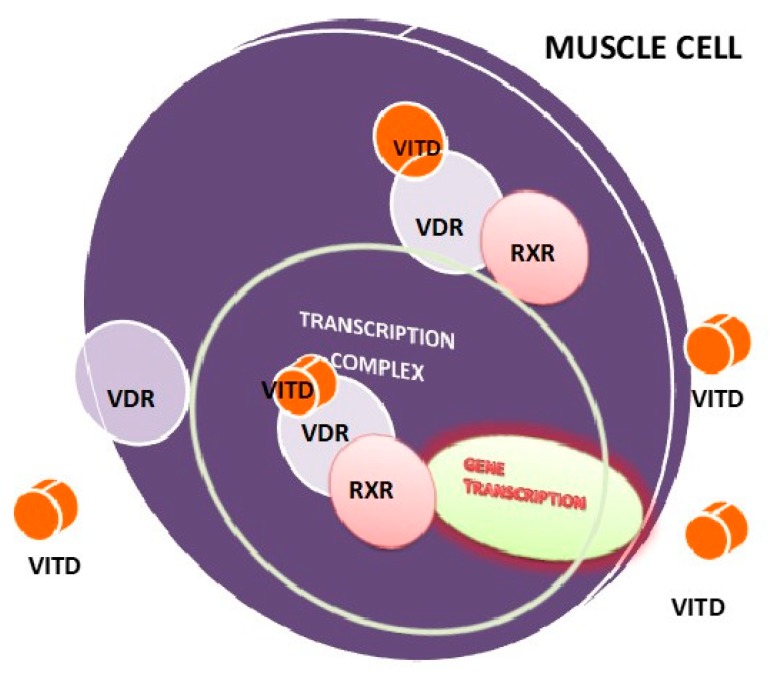
Proposed model for effect of vitamin D on skeletal muscle. Circulating and locally converted 1,25(OH)2D3 have been proposed to act on skeletal muscle through the vitamin D receptor (VDR). The VDR is located both within the nucleus, which results in genomic actions, and outside of the nucleus, which may cause acute nongenomic signaling events.

The VDR exerts its effects in two pathways:

- The first, the genomic pathway (slow or nuclear), through which the transcription and translation of the target genes are modified. This finding suggests that vitamin D promotes muscle cell proliferation and differentiation [15,20,60,61,62].
- The second mechanism is the non-transcriptional signaling pathway associated with the membrane (rapid, non-genomic or membrane), in which the receptor for 1.25-OHVITD is located. It has been shown that this mechanism enhances the interaction between myosin and actin in the sarcomere, making the force of muscle contraction stronger [11,15,20,57,63] (Figure 2).

In relation to physical exercise and its impact on athletes, it is argued that the low level of VITD could directly affect muscle strength and performance. Studies in young people and in elderly who are non-athletes found that low VITD levels were negatively associated with muscle strength markers [51,58]. For athletes with VITD deficiency, supplementation with the vitamin probably improves certain parameters of muscle performance [32,60]. In injured athletes, insufficient VITD also seems to delay rehabilitation and recovery after orthopedic surgery [49,63].

From a clinical point of view, a potential association between VITD and muscle function is also suggested, since myopathy was strongly associated with severe VITD deficiency [59]. Barker et al. found that 93% of the patients who presented common clinical symptoms of non-specific musculoskeletal pain had VITD deficiency.

The data presented above suggest that VITD could improve muscle mass and strength [59,63]. and could accelerate the recovery of muscles from the stress of intense exercise [60,61,62,63].

To conclude, it is suggested that vitamin D is beneficial for people as it increases the synthesis of muscle proteins, the concentration of adenosine triphosphate (ATP), strength, jump height, jumping speed and power, as well as the capacity to perform aerobic and anaerobic exercise. Physical performance could be significantly improved and/or preserved with adequate levels of vitamin D. Vitamin D also prevents muscle degeneration and reverses myalgia [63].

### 7.4. Effects of Vitamin D on Lung Function

Vitamin D insufficiency has been associated with impaired lung function, asthma and chronic obstructive pulmonary disease (COPD). On the other hand, vitamin D deficiency resulted in deficits in lung volume and correlated with multiple indices of compromised lung function and increased airway reactivity [64]. These actions of VITD favor alveolar structural integrity, pulmonary compliance, vital capacity and oxygen exchange [64,65,66,67]. 

Among the population of athletes, exercise performance and aerobic capacity (VO2max) depend on all these lung functions above. Adequate VO2max levels are needed in all sports activities. However, the results found for different authors in the athletic population in relation to VITD deficiency and athletes are inconclusive [64,65,66,67]

### 7.5. Vitamin D and Cardiovascular Function

#### Athlete’s Heart

First, we must bear in mind that the regular practice of intense physical exercise is associated with several structural and cardiac electrophysiological adaptations that improve diastolic filling and facilitate a sustained increase in cardiac output, which is essential for athletic performance. The vast majority of athletes show relatively slight structural and electrical changes, which are considered within the conventional definition of normal limits. Such cardiac adaptations are collectively known as “Athlete’s Heart” and are often reflected in the ECG and imaging studies [67].

Numerous factors influence the adaptations of athlete hearts, including sports modality, duration and intensity of training, age, ethnicity, gender, anthropometry and substance abuse to improve performance [68].

A small proportion of athletes develop pronounced changes that overlap with phenotypic expressions of heart disease involved in sudden cardiac death associated with exercise (SCD). In these circumstances, distinguishing between physiological adaptation and cardiac pathology is challenging, but a misdiagnosis can have serious consequences. Emerging studies suggest that ethnicity is a major determinant of cardiovascular adaptation to exercise, which should always be considered during the evaluation of an athlete. It is a well-established fact that ethnicity is one of the factors that influence the manifestations of an athlete’s heart [67,68,69].

It is also recognized that many professional athletes have vitamin D deficiency and, currently, no study has examined the association between vitamin D levels and the cardiac structure and function in healthy athletes. One thing to bear in mind is that recent research has detected an association between VITD and sudden cardiac death in athletes, finding a strong relationship between severe VITD deficiency and sudden cardiac death [67,68,69,70]. 

## 8. Mechanism of Action of Vitamin D in Cardiovascular Function

Vitamin D receptors (VDR) are present throughout the heart and vascular system, specifically located in myocytes and cardiac fibroblasts [1,9,20]. The activated form of VITD, 1-2OH VITD, participates in the structural remodeling of cardiac muscle and vascular tissue and activate myocyte contractility [57,59,61,63]. 

There is scientific evidence that vitamin D deficiency has long-term cardiovascular adverse effects. Vitamin D deficiency negatively affects cardiac contractility, vascular tone, cardiac collagen content and cardiac tissue maturation. This is mainly because VITD deficiency causes an increase in PTH levels that can lead to left ventricular hypertrophy. This hypertrophy can alter the filling capacity of the ventricle and ejection fraction leading to possible hypoxia of muscle tissue and a decrease in athletic performance [68,69,70]. It has also been evinced that in patients who presented a severe VITD deficiency, supplementation treatment resulted in an improvement in cardiac muscle function [68].

At the vascular level, there are vitamin D receptors in the vascular wall, so this vitamin is believed to affect vascular physiology and its pathophysiology [71,72,73,74]. Vitamin D insufficiency is related to increased arterial stiffness and endothelial dysfunction in blood vessels and promotes atherogenesis [70]. Severe vitamin D deficiency causes an alteration in the adaptive immune response favoring vascular dysfunction, insulin resistance and arteriosclerosis [67,68]. These factors are critical for aerobic and anaerobic exercise performance and resistance capacity [69]. Additionally, low serum levels of vitamin D can cause pathological myocardial hypertrophy, increased blood pressure and endothelial dysfunction. This confluence of alterations supports the assumption that inadequate levels of vitamin D could negatively influence cardiorespiratory capacity, influencing the supply of oxygen and nutrients to the exercising muscle.

Recent data has shown a high prevalence of vitamin D deficiency among ethnicities, particularly among Arab athletes. Vitamin D deficiency is associated with hypertension, myocardial infarction and stroke, as well as other diseases related to cardiovascular diseases. To date, the association between vitamin D levels, ethnicity and cardiovascular function in athletic populations has not been studied [53].

### 8.1. Action of Vitamin D in the Immune System

Different studies have proved that vitamin D affects innate and adaptive immunity through its VDR action [71,72,73]. Vitamin D affects both T and B cells. In resting conditions, the expression of VDRs shows low activity in both T and B cells, but in infectious diseases, they increase their activity, which suggests a crucial role in adaptive immunity [73].

Vitamin D can reduce inflammation by its inhibitory effect on proinflammatory cytokines such as interleukin-6, which converts monocytes into macrophages and produces more inflammatory cytokines [11,15,20]. Interleukin-6 can be early increased in intense training [71,72] and it is believed to be related to the appearance of muscle damage during training. On the other hand, it has been shown that vitamin D reduces the production of other proinflammatory cytokines such as interferon, interleukin-2 and tumor necrosis factor-6 [37,38,39,40]. Low levels of VITD in the general population and in athletes (especially after intense exercise) result in an increase in IL6 and TNFα. Therefore, vitamin D improves this inflammatory response [71,72,73].

By endorsing the above, the insufficiency of vitamin D in athletes is associated with a higher frequency of diseases, including common colds, influenza and gastroenteritis. In athletes, the incidence of respiratory diseases is higher (especially at the elite level), suggesting that low levels of vitamin D may favor the vulnerability of these professionals to upper respiratory tract infections, while individuals with higher levels of vitamin D show a lower propensity to them [65,66,73].

### 8.2. Effects of Vitamin D on the Nervous System

Vitamin D affects the central and peripheral nervous systems. Vitamin D receptors are present throughout the brain, including the primary motor cortex, which is the region that coordinates movement [9,11,74].

In turn, vitamin D also affects neuronal differentiation, maturation and growth. It also exerts direct neuroprotective effects through the synthesis of proteins that play a vital role in neural activity, including transmission. The GABAergic function is the main “brake” in the brain that affects muscle relaxation through corticospinal neurons [12,20,74,75]. The effects of vitamin D on GABAergic tone and on serotonin and dopamine are crucial for muscle coordination and for avoiding central fatigue, a condition associated with the synaptic concentration of several neurotransmitters. A high proportion of serotonin and dopamine affects exercise performance due to its effect on the general feeling of tiredness and perceptions of effort [12,15,17,76]. Another mechanism, through which vitamin D affects the brain and sports performance, may involve nociceptors, or rather, the sensory nerve cell that responds to noxious stimuli by sending signals to the spinal cord and brain. Nociceptors are full of VDR and 1α-hydroxylase. When these receptors transfer pain signals to the brain, an inhibitory physical response takes place. The relevance of this mechanism and physical activity/vitamin D is based on recent findings in animal studies that indicate that vitamin D depletion could result in hyperinnervation and nociceptive hypersensitivity in deep muscle tissue and loss of balance without affecting muscle strength or cutaneous nociceptive response [12,21,75]. Based on this finding, we could speculate that nociceptive hyperinnervation and hypersensitivity in deep muscle tissue could cause a false appearance of myalgia during physical activity that could reduce performance in individuals with vitamin D deficiency.

## 9. Supplementation with Vitamin D in Athletes

Despite the special attention given to the diet of athletes, we must bear in mind that some micronutrient deficiencies may appear. It is generally believed that if athletes follow a balanced diet, they will not require supplements [3,8,9,77,78,79,80]. However, this idea may be too simplistic. First, determining dietary fitness in athletes can be challenging. 

The micronutrient requirements of these professionals may vary depending on the duration, intensity and type of training [30,32,35,80,81,82]. Secondly, for some micronutrients, especially vitamin D, there may not be ample food sources. The importance of this issue lies in the fact that an athlete’s micronutrient status can affect their physical performance [15]. On the other hand, VITD activity is related to the adequate presence of other nutritional factors and it is very important to know the status of other nutrients, like magnesium. Magnesium plays an important role in bone mineralization due in part to its positive influence in the synthesis of active VITD. New research evidence that magnesium implementation can potentiate the effectiveness of VITD activity [79]. 

VITD deficiency leads to an increased risk of morbidity that could negatively influence athletic performance and significantly shorten the lifespan of professional athletes. Although some researchers have reported the improved effect of VITD supplementation on physical performance, the issue remains controversial [83,84,85,86,87,88,89,90,91,92].

Suboptimal VITD levels appear both in athletes who mainly train indoors, and at higher latitudes, and in those who train outdoors at lower latitudes [53,81,86]. We must remember that one of the factors that has the greatest influence on vitamin D levels is exposure to sunlight. Anything that limits the amount or quality of sun exposure, can compromise vitamin D levels [78,79,80]. 

Few published studies categorically state that vitamin D supplementation benefits neuromuscular and aerobic performance. In a recent randomized placebo-controlled trial, the effect of vitamin D (5000 IU per day over a period of eight weeks) on speed times and vertical jumps in a cohort of athletes was evaluated. The group that received vitamin D supplements recorded a substantial increase in vertical jump heights from the beginning to the end of the study period, while no change was observed in the placebo-controlled group [82].

Wyon et al. [90,91] found an improvement in neuromuscular performance in elite ballet dancers in a study of oral vitamin D3 supplementation. A significant increase in isometric strength (18.7%) and vertical jump (7.1%) was observed. The intervention group showed a significant decrease in the number of injuries with respect to the control group. However, other studies were unable to document any benefits following vitamin D supplementation in athletes with adequate or moderately deficient levels of vitamin D prior to supplementation. Close et al. examined the effects of vitamin D3 supplementation on serum concentrations of 25 [OH] D and on various exercise performance rates in athletes. At the start of the study, 57% of the participants were found to have VITD deficiency. However, despite the increase observed in serum levels of vitamin D, none of the groups showed an improvement in exercise performance compared to control ones [31,32]. Carswell et al. [85] in 967 young healthy military recruits found there was no influence of status VITD on muscular strength. Although supplementation restored VITD sufficiency, the beneficial effects on exercise performance remain unclear. However, they found a fairly positive association between VITD status and endurance performance. 

## 10. Supplementation with the Appropriate Dose of Vitamin D

It seems that vitamin D supplementation in the general population is important for preventing and avoiding its deficiency. However, there is a lot of controversy regarding the appropriate supplement doses. In athletes, it is even more controversial. 

Several vitamin D guidelines and guidance papers have been published with heterogeneous and partially opposed opinions and recommendations regarding vitamin D requirements [89,90,91,92].

Carlberg et al. suggest that a threshold level of VITD is not enough to asseverate the needs of VITD in individuals. The efficiency of the molecular response to VITD is critical for establishing the appropriate dose of VITD in each individual. This researcher’s evidence that VITD supplementation and his dose is related to the “personal vitamin D response Index” [88,91].

As stated above, the Institute of Medicine (IOM) concluded, in the 2011 consensus statement, that 25 (OH) D levels of 20 ng/mL (50 nmol/L) meet the needs of at least 97.5% of the (North American) population at all stages of life [47].

The Recommended Dietary Allowance (RDA) to meet the requirements of the IOM of vitamin D for the US and Canada is 600 IU for children and adults under 70 years of age and 800 IU for those over 70 years old. Although the US recommendation is higher than the one established in other countries, many VITD experts believe that these recommendations were established for bone health maintenance, but may not be sufficient to maintain non-skeletal benefits, as well as the optimal health and performance of athletes [3,48,52,77,86].

The Endocrinology Society estimated that 600–800 IU were not sufficient to ensure adequate levels and raised the recommended intake to 1500–2200 IU/day for individuals who do not have adequate sun exposure to maintain adequate vitamin D levels [3,5,8]. In relation to athletes, there is no evidence to suggest that their requirements are different from those of the general population.

A randomized controlled clinical trial in 70 athlete subjects was randomly divided into two groups, VITD supplementation and control. They found that weekly uptake of 50.000 IU VITD improved only certain athlete performance tests, and they conclude that the optimum dosage for athletes needs further studies [87].

Additionally, possible intoxications due to inadequate vitamin D supplementation should be taken into account. Vitamin D toxicity may be the result of the intake of excessive amounts of supplements of this vitamin. No cases of vitamin D toxicity have been reported from sunlight or regular food intake. The symptoms of vitamin D toxicity are produced by the resulting hypercalcemia which can lead to anorexia, frequent urination, excessive thirst, nausea, vomiting and, in severe cases, altered mental status and kidney failure. Many cases of vitamin D intoxication are the result of improperly manufactured supplements [83]. Some athletes and coaches live in the belief that “if a little is good, more is better”, which is a dangerous misconception. It is very important that the supplementation is carried out by professionals with knowledge on the subject and who are aware that, although VITD intoxication is very rare, it can occur. The most frequent cases are due to unintentional consumption of extremely high doses, and in many cases due to industrial error [3,5,8,83].

## 11. Conclusions 

The purpose of our review was to investigate the relevance of vitamin D in athletic performance. We review recent advances in this field and novel insights about vitamin D supplementation in athletes.

Low vitamin D status could negatively impact the health and training efficiency of athletes. Research to date suggests that certain athletes are at risk for suboptimal vitamin D status, which may increase risks for stress fractures, acute illness, and suboptimal muscle function. 

The emerging evidence about vitamin D and athletic performance suggests the need to determine vitamin D concentration in athletes but further research is necessary to characterize the true vitamin D status by simply measuring free vitamin D rather than total 25-OHVITD. 

In relation to the prevention of vitamin D deficiency, we must be aware that sun exposure is the main source. Unfortunately, there is evidence concerned about the possibility that sun exposure, if uncontrolled, may promote skin cancer. On the other hand, we must also take account nutrition in athletes and vitamin D. A personalized nutrition plan should develop. Sufficiency of essential minerals and micronutrients, like magnesium, are critical to enhancing activation of vitamin D. 

One interesting theory is based on individual molecular response to vitamin D. Athletes with personalized supplementation of vitamin D, will contribute to obtain optimized clinical benefits. Future studies could determine the optimal VITD threshold and determine supplementation recommendations. 

Although previous studies seem to suggest that VITD supplementation in athletes may have a beneficial effect on athletic performance, these results cannot be generalized. Unnecessary supplementation with high doses of vitamin D might be a relatively common practice, without proven benefit, and even with the risk of harm. 

Future research is needed, focusing on double-blinded supplementation and optimal VITD levels in athletes and to investigate VITD potentially positive influence on exercise performance and the benefits of VITD supplementation on athletic performance. 

## Figures and Tables

**Table 1:** Summarizes the thresholds for blood vitamin D (VITD) concentration stablish by the Institute of Medicine (IOM) and accepted by most organizations and researchers of vitamin D as well as its impact on people’s health.

Serum 25-Ohvitd Concentrations (nmol/L)		Health Status (ng/mL)	vitamin D Status
**< 30**	< 12	Associated with VITD deficiency, leading to rickets in infants and children and osteomalacia in adults	Severely Deficient
**30 to 50**	12 to 20	Generally considered inadequate for one and overall health in healthy individuals	Deficient/ Insufficient
**50 to 125**	20 to 50	Generally considered adequate for bone and overall health in healthy individuals	Adequate
**> 125**	> 50	Emerging evidence links potential adverse effects to such high levels, particularly >150 nmol/L (>60 ng/mL)	Inadequate/ Toxic
